# Multicenter study evaluating one multiplex RT-PCR assay to detect SARS-CoV-2, influenza A/B, and respiratory syncytia virus using the LabTurbo AIO open platform: epidemiological features, automated sample-to-result, and high-throughput testing

**DOI:** 10.18632/aging.203761

**Published:** 2021-12-12

**Authors:** Hsing-Yi Chung, Ming-Jr Jian, Chih-Kai Chang, Jung-Chung Lin, Kuo-Ming Yeh, Ya-Sung Yang, Chien-Wen Chen, Shan-Shan Hsieh, Sheng-Hui Tang, Cherng-Lih Perng, Feng-Yee Chang, Kuo-Sheng Hung, En-Sung Chen, Mei-Hsiu Yang, Hung-Sheng Shang

**Affiliations:** 1 Division of Clinical Pathology, Department of Pathology, Tri-Service General Hospital, National Defense Medical Center, Taipei, Taiwan, ROC; 2 Division of Infectious Diseases and Tropical Medicine, Department of Medicine, Tri-Service General Hospital, National Defense Medical Center, Taipei, Taiwan, ROC; 3 Division of Pulmonary and Critical Care Medicine, Department of Medicine, Tri-Service General Hospital, National Defense Medical Center, Taipei, Taiwan, ROC; 4 Center for Precision Medicine and Genomics, Tri-Service General Hospital, National Defense Medical Center, Taipei, Taiwan, ROC; 5 Department of Clinical Pathology, Cathay General Hospital, Taipei, Taiwan, ROC

**Keywords:** COVID-19, SARS-CoV-2, B.1.1.7 variant, multiplex testing, SARS-CoV-2 VOC

## Abstract

Since the Coronavirus 19 (COVID-19) pandemic, several SARS-CoV-2 variants of concern (SARS-CoV-2 VOC) have been reported. The B.1.1.7 variant has been associated with increased mortality and transmission risk. Furthermore, cluster and possible co-infection cases could occur in the next influenza season or COVID-19 pandemic wave, warranting efficient diagnosis and treatment decision making. Here, we aimed to detect SARS-CoV-2 and other common respiratory viruses using multiplex RT-PCR developed on the LabTurbo AIO 48 open system. We performed a multicenter study to evaluate the performance and analytical sensitivity of the LabTurbo AIO 48 system for SARS-CoV-2, influenza A/B, and respiratory syncytial virus (RSV) using 652 nasopharyngeal swab clinical samples from patients. The LabTurbo AIO 48 system demonstrated a sensitivity of 9.4 copies/per PCR for *N2* of SARS-CoV-2; 24 copies/per PCR for *M* of influenza A and B; and 24 copies/per PCR for *N* of RSV. The assay presented consistent performance in the multicenter study. The multiplex RT-PCR applied on the LabTurbo AIO 48 open platform provided highly sensitive, robust, and accurate results and enabled high-throughput detection of B.1.1.7, influenza A/B, and RSV with short turnaround times. Therefore, this automated molecular diagnostic assay could enable streamlined testing if COVID-19 becomes a seasonal disease.

## INTRODUCTION

Severe acute respiratory syndrome coronavirus 2 (SARS-CoV-2), the causative agent of coronavirus disease 2019 (COVID-19), has spread worldwide, with over 180 million confirmed cases of infection (https://covid19.who.int/ accessed: 2021/07/06). The B.1.1.7 variant is estimated to have emerged in September 2020 and has quickly become the dominant circulating SARS-CoV-2 variant in England [1, 2]. Taiwan faced the third outbreak of COVID-19 just before the third week of April and is still ongoing. This third outbreak involved the alpha variant (B.1.1.7) [3, 4].

Currently, real-time reverse transcription-polymerase chain reaction (rRT-PCR) is the gold standard assay for early diagnosis in patients with suspected SARS-CoV-2 infection [5–7]. However, some studies have showed that targeting a particular detection region might result in the loss of sensitivity for various SARS-CoV-2 variants [8]. In addition, it is sometimes difficult to distinguish COVID-19 from other respiratory illnesses caused by other respiratory viruses because of common clinical manifestations including fever, cough, and dyspnea [9–12]. Consequently, SARS-CoV-2 and other upper respiratory viruses, including influenza virus and respiratory syncytial virus (RSV), have to be concurrently tested by rRT-PCR in symptomatic patients.

Several commercial kits support the rapid detection of multiple pathogens, including SARS-CoV-2, influenza A/B, and other respiratory pathogens [13]. However, these kits were not affordable cost and could not offer high throughput during the COVID-19 pandemic in developed countries. In addition, in light of the high demand for nucleic acid-amplification tests, continuing shortage of supplies, and high sensitivity of molecular diagnostics, there is a need for one multiplex assay to simultaneously screen all four viruses (SARS-CoV-2, influenza A, B, and RSV) with the same reaction [14].

In this study, we developed a sample-to-result platform that fully automated laboratory-developed multiplex RT-PCR assay to simultaneously detect SARS-CoV-2, influenza A/B, and RSV in one tube on the LabTurbo AIO 48 system.

## RESULTS

### Epidemiological features

We retrieved the clinical dataset of 102 patients infected by the SARS-CoV-2 B.1.1.7 variant. We analyzed RNA extracted from positive specimens using VirSNiP SARS-CoV-2 Spike N501Y and Spike del H69/V70 (TIB Molbiol, Berlin, Germany) to confirm the variant type. All data of all patients were reported between May 1 and July 4, 2021. We analyzed age distribution of the SARS-CoV-2 (B.1.1.7)-positive patients in our dataset (Table 1) and found that the youngest and oldest non-survivors died at 43 and 91 years of age, respectively. Elderly patients ≥70 years accounted for only 59% of the 22 survivors. Our sex analysis showed that COVID-19-related deaths were more among elderly males (50%, total 14) than among younger males (15%, total 40). Female patients showed a similar proportion of non-survivors, with mortality of 30.0% in elderly females and 5.3% in younger females (Table 2).

**Table 1:** Clinical features of COVID-19 patients with the symptoms and clinical outcomes.

**Characteristics**	**SARS-CoV-2 (B.1.1.7)**
**Total number**	102
**Gender**	
Male	54
Female	48
**Age**	
<19	2
20–49	28
50–69	47
>70	25
Mean	62.6
Medium	64
**Symptoms**	
Fever	76
Cough	83
Difficulty breathing	30
Burnout	7
Diarrhea	17
**Clinical outcome**	
Survivors	84
Non-survivors	18

**Table 2:** Basic information of 102 SARS-CoV-2 (B.1.1.7) patients in our study.

	**Age**	**Survivors**	**Non-Survivors**
Male			
	Younger (<70)	34 (85.0%)	6 (15.0%)
	Elder (>70)	7 (50.0%)	7 (50.0%)
Female			
	Younger (<70)	36 (94.7%)	2 (5.3%)
	Elder (>70)	7 (70.0%)	3 (30.0%)
Total		84	18

### Analytical sensitivity of the one multiplex rRT-PCR on the LabTurbo AIO 48 open platform

To validate the sensitivity of the designed multiplex, we tested all these pathogens (SARS-CoV-2, influenza A virus (subtypes H1, H1N1, and H3), influenza B virus, and RSV (subtypes A and B)) in one multiplex RT-PCR on the AIO48 open system. We tested several dilutions to determine the LoD by repeating the experiments 20 times. We defined limit of detection (LoD) as the minimum concentration with a positive detection rate of 95%. The LoD for each target was 9.4 copies/PCR reaction for the *N2* gene of SARS-CoV-2; the LoD for influenza A/B virus (*M* gene) and RSV (*N* gene) reached 24 copies/PCR (Table 3). A mixed RNA sample was also tested using the same protocol. The LoD was the same as the above (Supplementary Table 1). Additionally, there was no cross-reaction among the respiratory pathogens.

**Table 3:** Assessment of Limit of detection for SARS-CoV-2, influenza A/B, RSV in multiplex RT-PCR.

**Pathogen**	**Gene target/fluorescent dye**	**No. of replicates detected at each dilution/total no. of replicates at indicated no. of copies per PCR (percentage)**				
		**300**	**75**	**24**	**18.8**	**9.4**
SARS-CoV-2	*N2*/FAM	20/20 (100)	20/20 (100)	20/20 (100)	20/20 (100)	19/20 (95)
Influenza A H1	*M*/VIC	20/20 (100)	20/20 (100)	20/20 (100)	16/20 (80)	N.D.
Influenza A H3		20/20 (100)	20/20 (100)	20/20 (100)	9/20 (45)	N.D.
Influenza A H1N1		20/20 (100)	20/20 (100)	20/20 (100)	16/20 (80)	N.D.
Influenza B	*M*/Cy5	20/20 (100)	20/20 (100)	20/20 (100)	10/20 (50)	N.D.
RSV subtype A	*N*/Cy5.5	20/20 (100)	20/20 (100)	20/20 (100)	12/20 (60)	N.D.
RSV subtype B		20/20 (100)	20/20 (100)	20/20 (100)	8/20 (40)	N.D.

N.D. is not detected.

### Analytical specificity of the one multiplex rRT-PCR on the LabTurbo AIO 48 platform

We tested the analytical specificity of the lab-developed multiplex PCR test performed on the LabTurbo AIO 48 system for upper respiratory viruses other than SARS-CoV-2, influenza A/B virus, and RSV. Clinical samples or cell supernatants positive for rhinovirus/enterovirus, parainfluenza virus, cytomegalovirus, herpes simplex virus, varicella-zoster virus, and adenovirus were obtained from the Taiwan CDC Viral Infection Contract Laboratory. There was no cross-reactivity among these organisms (Table 4).

**Table 4:** The cross-reactivity tests of multiple RT-PCR from clinical samples or cell supernatants.

**Pathogen**	**Gene targets of multiple rRT-PCR (positive no./total test no.)**			**Final performance**
	***N2* gene**	***M* gene**	***N* gene**	
Parainfluenza virus	0/3	0/3	0/3	N.D.
Rhinovirus/Enterovirus	0/2	0/2	0/2	N.D.
Varicella-Zoster Virus	0/5	0/5	0/5	N.D.
Cytomegalovirus	0/5	0/5	0/5	N.D.
Herpes simplex virus type1 type 1	0/5	0/5	0/5	N.D.
Adenovirus	0/5	0/5	0/5	N.D.

N.D. is not detected.

### Clinical performance of the multiplex rRT-PCR on the LabTurbo AIO 48 system platform

**Figure 1:**
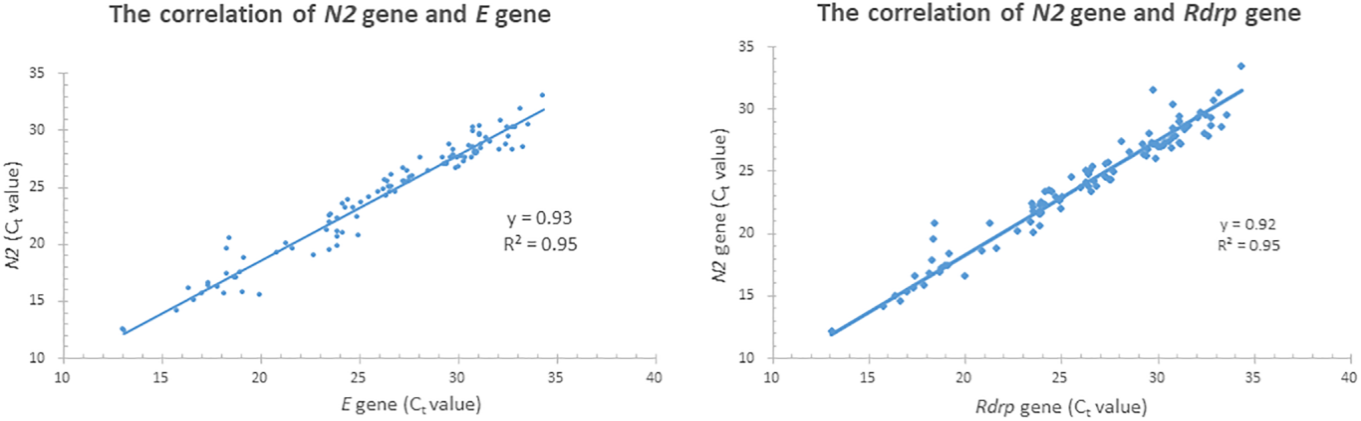
The correlation between *N2*, *E* and *Rdrp* gene of 102 SARS-CoV-2 positive specimens.

**Figure 2:**
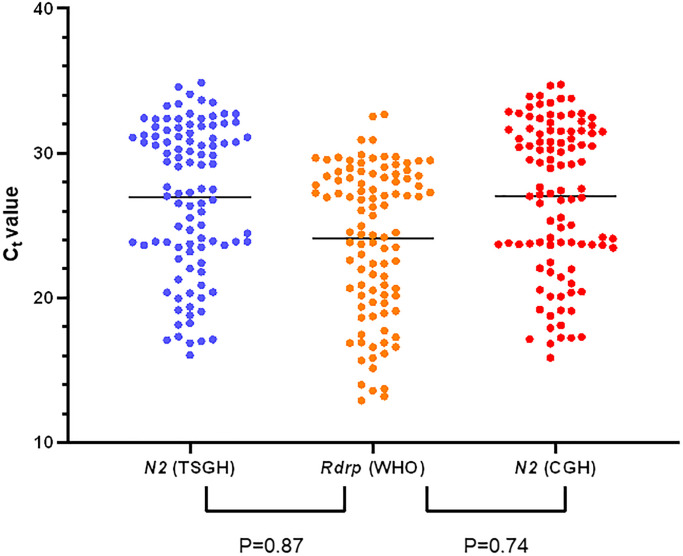
The clinical performance of multiplex RT-PCR in SARS-CoV-2 positive specimens between the two medical centers.

We analyzed 652 retrospective specimens in this study: 102 from SARS-CoV-2-positive patients and 550 from SARS-CoV-2-negative patients. The *N2* gene from the multiplex rRT-PCR mixture and *RdRp* and *E* genes recommended by the WHO guidelines were compared for all positive specimens. Figure 1 shows a high correlation between the *N2* and *Rdrp* genes (R^2^ = 0.95). Similar results were obtained for the *N2* and *E* genes (R^2^ = 0.95). There was no apparent difference between those targets and no false positive nor negative result between those positive samples. Among the positive specimens, 102 specimens from SARS-CoV-2-positive patients were tested at two medical centers (Tri-Service General Hospital (TSGH) and Cathay General Hospital (CGH)) independently, followed by the same multiplex on the LabTurbo AIO platform. Those selected 102 samples with the Ct level that satisfied the range for high to low SARS-CoV-2 loads. Figure 2 shows our assay targeting the *N2* gene of SARS-CoV-2 without a significant difference in the results between the two medical centers. Furthermore, there were no false-positives or false-negatives among these specimens. The results of the two centers presented 100% agreement with each other.

### Results of co-infection of SARS-CoV-2 and other respiratory pathogens

We analyzed 652 specimens tested for SARS-CoV-2 and other respiratory pathogens using our multiplex rRT-PCR on the AIO LabTurbo open platform. The 102 SARS-CoV-2-positive specimens were found to be positive for SARS-CoV-2, whereas none was found to be positive for one or more non–SARS-CoV-2 pathogen(s). Among the tested specimens, influenza A virus was the most commonly detected pathogen (*n* = 19), followed by influenza B virus (*n* = 5) and RSV (*n* = 10). This finding highlighted the importance of differentiating other causes of respiratory illness from SARS-CoV-2.

## DISCUSSION

The SARS-CoV-2 B.1.1.7 variant of concern (VOC), which was first detected in South-East England, is more transmissible than previously circulating variants [15]. Currently, it accounts for 50–90% of the COVID-19 cases in the US and Europe and spreading over 170 countries [16]. During the COVID-19 pandemic, SARS-CoV-2 B.1.1.7 has been associated with increased secondary attack rate [17], and risk of hospitalization, severity, and mortality [18, 19]. In the present study, we used specimens from 102 individuals with COVID-19 between May 2021 and July 2021. Non-Survivors comprised 50% of the total elder males (>70 years old) and 30% of the total elderly females. Currently, Taiwan is facing the third wave of SARS-CoV-2 B.1.1.7 infection.

Our multiplex RT-PCR assay on the LabTurbo AIO 48 open platform showed good performance. COVID-19 symptoms are similar to flu-like symptoms. The flu-like symptoms include fever, chills, headache, muscle or body aches, cough, sore throat, runny nose, fatigue, nausea, vomiting, and diarrhea, and they are caused by different respiratory tract pathogens. The major respiratory tract pathogens include influenza A and B, RSV, adenovirus, enterovirus, human metapneumovirus, parainfluenza virus, adenovirus, rhinovirus, and human coronavirus. Hence, the pathogens causing these symptoms will be difficult to distinguish in the next flu season [20]. The death rate associated with different viruses varies worldwide. The death rate of patients infected with influenza virus could reach 250,000–500,000 individuals worldwide. RSV is associated with an estimated 132,000–172,000 pediatric hospitalizations in the United States annually. Since 2020, SARS-CoV-2 infection has resulted in over four million deaths. In addition, 3% of patients with COVID-19 are co-infected by other respiratory tract viruses. Influenza A virus and RSV were the top two pathogens, which accounted for 30% of viral co-infections [21]. Recently, several companies have developed commercial kits to detect SARS-CoV-2, influenza virus, and/or RSV, such as Liat SARS-CoV-2 and Influenza A/B (Roche Molecular Systems, Inc., Pleasanton, CA, USA), Cepheid Xpert Xpress SARS-CoV-2/Flu/RSV (Cepheid, Sunnyvale, CA, USA), and BioFire Respiratory Panel 2.1 (RP2.1; BioFire Diagnostics, LLC, Salt Lake City, UT, USA). However, these molecular diagnostic tests rely on specific platforms with specific reagent requirements. That turnaround time of these commercial kit is approximately 25–45 min for one sample. The turnaround time increases with the number of specimens. For example, in BioFire Respiratory Panel 2.1, 48 specimens can be tested in 36 h with one machine. The capacity of the multiplex RT-PCR assay that we developed could reach approximately 2 h for 48 samples. It will decrease the TAT by three-fold for 48 samples. Thus, our assay is a valuable tool with a better efficacy and turnaround time for the simultaneous detection of SARS-CoV-2, influenza A virus, influenza B virus, and RSV than commercial kits. Our study provides a perspective to decide which molecular diagnostic test to implement in clinical laboratories. Our assay could accurately identify SARS-CoV-2 and other common respiratory viral infections. Simultaneous testing of all four pathogens shortens the turnaround time and could thus increase the effectiveness of control and prevention measures by health providers and departments. Infections with common respiratory viruses are associated with similar symptoms that are not easy to distinguish from each other. Furthermore, in patients with COVID-19, a pooled proportion meta-analysis has shown that 3% of patients were co-infected with other viruses [21]. Hence, in the next flu season, the multiplex RT-PCR might be a valuable tool to distinguish pathogens.

To diagnose RNA virus infections, RT-PCR is the most common method owing to its accuracy and popularity [22]. Currently, numerous primers have been designed to target various RNA sequences in six genes of SARS-CoV-2 for diagnostic purposes, including *ORF*1a/b, *RdRp* (RNA-dependent RNA polymerase), *S* (spike protein), *E* (envelope), and *N1/N2/N3* (nucleocapsid). Among these, the nucleocapsid *N2* and envelope *E* genes can be most sensitively detected, as described previously [23]. Here, we targeted the *N2* gene of SARS-CoV-2 and demonstrated that the performance of our method in detecting the *N2* gene was as good as that for detecting the *E* and *RdRp* genes, which are targeted by the WHO protocol. There was no apparent difference between the two targets and there is no false positive nor negative result between those positive samples. Hence, this multiplex could afford a good performance in preventing the spread of COVID-19.

However, our study has some limitations. First, the number of positive cases was small, as the number of initially confirmed COVID-19 cases in Taiwan was approximately 1,000. Thus, studies with a higher number of positive cases are required in the future. Furthermore, to assess the clinical performance of the LabTurbo AIO 48 system in detecting common upper respiratory viral pathogens, including SARS-CoV-2, we enrolled two medical centers. Our concern regarding this approach was whether the initial challenges encountered during the management of patients with COVID-19 potentially decreased the number of requests for virus culture tests to rule out other infections. Nevertheless, we used different instruments from the two medical centers to verify the same specimen, with consistent results. Second, this multiplex reagent just provides one *N2* gene for SARS-CoV-2. This was a screen test for pathogens infecting the upper respiratory tract. According to the Taiwan Centers for Disease Control guideline, we should retest the positive specimen in other genes. Hence, we suggest the specimens should be confirmed by other genes (for example, *E*, *Rdrp*, *N1*, *N3*, and *ORF1ab*). Furthermore, at US CDC, the *N2* gene was the confirmed target for the SARS-CoV-2 positive specimens.

Despite these limitations, there are several advantages of using our LDT multiplex RT-PCR assay to detect both SAR-CoV-2 and other upper respiratory pathogens. We used the LabTurbo AIO 48 system as a sample-to-result open platform, that is, from RNA extraction to nucleic acid amplification. The LabTurbo AIO system was combined with RNA extraction and RT-PCR thermocyclers. This could improve the robustness of extracted RNA to prepared master mix containing the desired primer and probe. Hence, the multiplex rRT-PCR we developed in this study could be applicable in other real-time PCR thermocyclers. We believe that this assay might be applies to other real-time PCR machines or sample-to-result platforms with open channels or open systems. Additionally, the multiplex PCR assay described here might serve as an alternative tool in clinical diagnostic laboratories for routine SARS-CoV-2 and influenza virus detection in the future when SARS-CoV-2 might gradually evolve into an endemic flu-like virus. Moreover, using the same reaction to detect SARS-CoV-2, influenza A/B virus, and RSV might help overcome the problem of shortage of supplies for nucleic acid extraction and PCR diagnostic reagents/equipment during the current COVID-19 pandemic.

## METHODS

### Specimen collection

Clinical upper respiratory samples were collected from May 1 to July 4, 2021. The retrospective specimens contained 102 SARS-CoV-2 positive and 550 negative samples, which were also confirmed using the WHO protocol described previously [24]. The *E* and *Rdrp* genes were confirmed in all positive samples by the central laboratory of the Taiwan Centers for Disease Control and Prevention, as reference data. This study was registered on March 20, 2021 and approved by the TSGH Institutional Review Board (approval number: C202005041). We tested all 652 nasopharyngeal swab specimens collected from patients suspected of having COVID-19, using LIBO Specimen Collection and Transport Swab Kits with Universal Transport Medium (New Taipei City, Taiwan). Influenza A and B- and RSV-positive specimens were confirmed using the BioFire^®^ respiratory panel 2.1 (RP2.1) assay. The same specimens were detected by two clinical laboratories: TSGH (Taipei City, Taiwan) and CGH (Taipei City) using the same protocol and platform.

### Assessment using the multiplex assay on the LabTurbo AIO 48 open system

In this study, we designed a multiplex PCR test that was performed on the LabTurbo AIO 48 system using specific primers and probes to simultaneously detect SARS-CoV-2, influenza A/B virus, and RSV in a well. The total viral nucleic acid was extracted from each swab in a universal viral transport medium (500 μL) to a final eluate volume of 60 μL using the LabTurbo Virus Mini Kit (Cat. No. LVN48-300) and an automated LabTurbo AIO open system. The Luna^®^ Probe One-Step RT-qPCR Kit (No ROX, New England Biolabs), comprising reverse transcriptase and 2× PCR master mix, was used according to the RNA testing kit instructions. For analysis on the LabTurbo AIO open system, each 25-μL reaction mixture contained 12.5 μL of 2× PCR master mix, 4 μL of primer/probe mixture, 1.25 μL of reverse transcriptase, 1.25 μL of RNase-free water, and 6 μL of extracted RNA. SARS-CoV-2, influenza A/B virus, and RSV were detected using the following thermal cycling conditions: 50°C for 10 min, 95°C for 2 min, and 45 cycles at 95°C for 10 s, 55°C for 25 s, and 64°C for 32 s. Here, we detected the *N2* gene in the SARS-CoV-2 genome, *M* gene in influenza A/B virus, *N* gene in RSV, and human ribonuclease *P* gene (RP) [25], which was also included as an internal control (Supplementary Table 2).

### Assessment of analytical sensitivity

To validate the sensitivity of the designed multiplex assay, we tested all these pathogens (SARS-CoV-2, influenza A (subtypes H1, H1N1, and H3), influenza B, and RSV (subtypes A and B)) in one multiplex RT-PCR on the AIO48 open system. RNA controls (Vircell, Granada, Spain) of known concentrations were used to prepare several dilutions to determine the LoD by repeating the experiments 20 times. We defined limit of detection (LoD) as the minimum concentration with a positive detection rate of 95%. The analytical sensitivity of the LabTurbo AIO 48 tests was defined as the lowest dilution at which all replicates were identified as positive (C_t_ < 35) for SARS-CoV-2.

### Evaluation of specificity

The specificity of multiplex master mix on the LabTurbo AIO platform was evaluated using viral cultures from the Taiwan CDC Viral Infection Contract Laboratory. We tested some common respiratory viruses (such as parainfluenza virus and enterovirus) to ensure that the master mix designed in this study could distinguish the virus of interest.

### Comparison of the performance using clinical specimens

To validate the performance of the multiple PCR assay designed in this study to detect SARS-CoV-2 (*N2* gene), all specimens were subjected to rRT-PCR again for the *E* and *Rdrp* genes, per the WHO panel. The original samples were confirmed those genes as the reference genes in TSGH. The same specimens were detected by two medical centers: TSGH and CGH using the same protocol on the LabTurbo AIO48 open platform. The C_t_ value of <35 was defined as a positive result for the pathogen. Each sample had an internal control (*Rnase P* gene). The external control comprised RNA spike-in mix as the positive control and H_2_O as the negative control.

### Whole-genome sequencing of SARS-CoV-2

Ovation RNA-Seq System V2 (Nugen Technologies, San Carlos, CA, USA) was used to synthesize cDNA, which was then processed into a library as described previously [26]. WGS was performed as described previously [27]. Briefly, whole-genome sequences of the SARS-CoV-2 isolates (TSGH-42 and TSGH-43) were obtained following the protocol of the Illumina TruSeq Stranded mRNA Library Prep Kit to enrich SARS-CoV-2 cDNA using multiplex RT-PCR amplicons. Next-generation sequencing was performed on the NovaSeq 6000 platform (Illumina, San Diego, CA, USA). Paired-end read assemblies of the whole virus genome sequence were formed using SPAdes assembler with SARS-CoV-2 isolate Wuhan-Hu-1, complete genome (NC_045512.2) to run the genome-guide assembly pipeline.

### Phylogenetic relationship analysis

To identify pathogen evolution relationships, assembly sequences were uploaded to the Nextclade website (https://clades.nextstrain.org/) developed by Nextstrain [28]. TSGH sequences were aligned to the reference sequences of major clades of SARS-CoV (20I) grouped using Nextstrain and phylogenetic tree annotated with these alignment definitions, using lists of grouped clade-defining mutations via Augur workflow [29] supported by Nextclade. The results are shown in Supplementary Figure 1.

### Classification of SARS-CoV-2 variant from positive specimen

To identify the variant type of SARS-CoV-2-positive specimens, we used the commercial kit developed by TIB Molbiol (Berlin, Germany). This kit can be used to rapidly detect SARS-CoV-2 VOC using extracted RNA with VirSNiP SARS-CoV-2 Spike N501Y and Spike del H69/V70. It was used to detect the mutations of SARS-CoV-2 using real-time RT-PCR post-melting curve analysis on LightCycler 480 (Roche Molecular Systems, Inc.) according to the manufacturer’s instructions. The results were read following the manufacturer’s instructions. The results were consistent with those of the rapid detection of SARS-CoV-2 variant.

## Supplementary Materials

Supplementary Figure 1

Supplementary Tables

## References

[1] Kirby T. New variant of SARS-CoV-2 in UK causes surge of COVID-19. Lancet Respir Med. 2021; 9:e20–21. 10.1016/S2213-2600(21)00005-9 33417829 PMC7784534

[2] Kidd M, Richter A, Best A, Cumley N, Mirza J, Percival B, Mayhew M, Megram O, Ashford F, White T, Moles-Garcia E, Crawford L, Bosworth A, et al. S-Variant SARS-CoV-2 Lineage B1.1.7 Is Associated With Significantly Higher Viral Load in Samples Tested by TaqPath Polymerase Chain Reaction. J Infect Dis. 2021; 223:1666–70. 10.1093/infdis/jiab082 33580259 PMC7928763

[3] Nordling TEM, Wu YH. Taiwan on track to end third COVID-19 community outbreak. medRxiv. 2021. [Preprint]. 10.1101/2021.06.20.21259178

[4] Hsu CY, Wang JT, Huang KC, Fan AC, Yeh YP, Chen SL. Household transmission but without the community-acquired outbreak of COVID-19 in Taiwan. J Formos Med Assoc. 2021 (Suppl 1); 120:S38–45. 10.1016/j.jfma.2021.04.021 33994234 PMC8092621

[5] Perng CL, Jian MJ, Chang CK, Lin JC, Yeh KM, Chen CW, Chiu SK, Chung HY, Wang YH, Liao SJ, Li SY, Hsieh SS, Tsai SH, et al. Novel rapid identification of Severe Acute Respiratory Syndrome Coronavirus 2 (SARS-CoV-2) by real-time RT-PCR using BD Max Open System in Taiwan. PeerJ. 2020; 8:e9318. 10.7717/peerj.9318 32596046 PMC7305773

[6] Avadhanula V, Nicholson EG, Ferlic-Stark L, Piedra FA, Blunck BN, Fragoso S, Bond NL, Santarcangelo PL, Ye X, McBride TJ, Aideyan LO, Patel KD, Maurer L, et al. Viral Load of Severe Acute Respiratory Syndrome Coronavirus 2 in Adults During the First and Second Wave of Coronavirus Disease 2019 Pandemic in Houston, Texas: The Potential of the Superspreader. J Infect Dis. 2021; 223:1528–37. 10.1093/infdis/jiab097 33585934 PMC7928726

[7] Mancini F, Barbanti F, Scaturro M, Fontana S, Di Martino A, Marsili G, Puzelli S, Calzoletti L, Facchini M, Di Mario G, Fabiani C, Bella A, Riccardo F, et al, and Istituto Superiore di Sanità (ISS) COVID-19 Team. Multiplex Real-Time Reverse-Transcription Polymerase Chain Reaction Assays for Diagnostic Testing of Severe Acute Respiratory Syndrome Coronavirus 2 and Seasonal Influenza Viruses: A Challenge of the Phase 3 Pandemic Setting. J Infect Dis. 2021; 223:765–74. 10.1093/infdis/jiaa658 33080031 PMC7665649

[8] Wollschläger P, Todt D, Gerlitz N, Pfaender S, Bollinger T, Sing A, Dangel A, Ackermann N, Korn K, Ensser A, Steinmann E, Buhl M, Steinmann J. SARS-CoV-2 N gene dropout and N gene Ct value shift as indicator for the presence of B.1.1.7 lineage in a commercial multiplex PCR assay. Clin Microbiol Infect. 2021; 27:1353.e1–1353.e5. 10.1016/j.cmi.2021.05.025 34044153 PMC8142743

[9] Wu X, Cai Y, Huang X, Yu X, Zhao L, Wang F, Li Q, Gu S, Xu T, Li Y, Lu B, Zhan Q. Co-infection with SARS-CoV-2 and Influenza A Virus in Patient with Pneumonia, China. Emerg Infect Dis. 2020; 26:1324–26. 10.3201/eid2606.200299 32160148 PMC7258479

[10] Chen Z, Li X, Li J, Zhang S, Zhou P, Yu X, Ren Y, Wang J, Zhang L, Li Y, Wu B, Hou Y, Zhang K, et al. A COVID-19 risk score combining chest CT radiomics and clinical characteristics to differentiate COVID-19 pneumonia from other viral pneumonias. Aging (Albany NY). 2021; 13:9186–224. 10.18632/aging.202735 33713401 PMC8064216

[11] Tan X, Zhang S, Xu J, Zhou M, Huang Q, Duan L, Lv Z, Xia H, Xiao W, Yin Z, Jin Y. Comparison of clinical characteristics among younger and elderly deceased patients with COVID-19: a retrospective study. Aging (Albany NY). 2020; 13:16–26. 10.18632/aging.202139 33323556 PMC7835042

[12] Wang W, He D, Chen J, Zhang Z, Wang S, Jiang Y, Wei J. Correction for: Circular RNA Plek promotes fibrogenic activation by regulating the miR-135b-5p/TGF-βR1 axis after spinal cord injury. Aging (Albany NY). 2021; 13:16897. 10.18632/aging.203271 34192667 PMC8266310

[13] Leung EC, Chow VC, Lee MK, Tang KP, Li DK, Lai RW. Evaluation of the Xpert Xpress SARS-CoV-2/Flu/RSV Assay for Simultaneous Detection of SARS-CoV-2, Influenza A and B Viruses, and Respiratory Syncytial Virus in Nasopharyngeal Specimens. J Clin Microbiol. 2021; 59:e02965–20. 10.1128/JCM.02965-20 33436456 PMC8092745

[14] Chung HY, Jian MJ, Chang CK, Lin JC, Yeh KM, Chen CW, Chiu SK, Wang YH, Liao SJ, Li SY, Hsieh SS, Tsai SH, Perng CL, et al. Novel dual multiplex real-time RT-PCR assays for the rapid detection of SARS-CoV-2, influenza A/B, and respiratory syncytial virus using the BD MAX open system. Emerg Microbes Infect. 2021; 10:161–66. 10.1080/22221751.2021.1873073 33410371 PMC7832498

[15] Davies NG, Abbott S, Barnard RC, Jarvis CI, Kucharski AJ, Munday JD, Pearson CAB, Russell TW, Tully DC, Washburne AD, Wenseleers T, Gimma A, Waites W, et al, and CMMID COVID-19 Working Group, and COVID-19 Genomics UK (COG-UK) Consortium. Estimated transmissibility and impact of SARS-CoV-2 lineage B.1.1.7 in England. Science. 2021; 372:eabg3055. 10.1126/science.abg3055 33658326 PMC8128288

[16] Shen L, Bard JD, Triche TJ, Judkins AR, Biegel JA, Gai X. Rapidly emerging SARS-CoV-2 B.1.1.7 sub-lineage in the United States of America with spike protein D178H and membrane protein V70L mutations. Emerg Microbes Infect. 2021; 10:1293–99. 10.1080/22221751.2021.1943540 34125658 PMC8238060

[17] Buchan SA, Tibebu S, Daneman N, Whelan M, Vanniyasingam T, Murti M, Brown KA. Increased household secondary attacks rates with Variant of Concern SARS-CoV-2 index cases. Clin Infect Dis. 2021. [Epub ahead of print]. 10.1093/cid/ciab496 34105720 PMC8384411

[18] Jassat W, Mudara C, Ozougwu L, Tempia S, Blumberg L, Davies MA, Pillay Y, Carter T, Morewane R, Wolmarans M, von Gottberg A, Bhiman JN, Walaza S, et al, and DATCOV Author Group. Increased mortality among individuals hospitalised with COVID-19 during the second wave in South Africa. medRxiv. 2021. [Preprint]. 10.1101/2021.03.09.21253184

[19] Davies NG, Jarvis CI, Edmunds WJ, Jewell NP, Diaz-Ordaz K, Keogh RH, and CMMID COVID-19 Working Group. Increased mortality in community-tested cases of SARS-CoV-2 lineage B.1.1.7. Nature. 2021; 593:270–74. 10.1038/s41586-021-03426-1 33723411 PMC9170116

[20] Sockrider M, Jamil S, Santhosh L, Carlos WG. COVID-19 Infection versus Influenza (Flu) and Other Respiratory Illnesses. Am J Respir Crit Care Med. 2020; 202:P27–28. 10.1164/rccm.2020C16 33021812

[21] Lansbury L, Lim B, Baskaran V, Lim WS. Co-infections in people with COVID-19: a systematic review and meta-analysis. J Infect. 2020; 81:266–75. 10.1016/j.jinf.2020.05.046 32473235 PMC7255350

[22] Abduljalil JM. Laboratory diagnosis of SARS-CoV-2: available approaches and limitations. New Microbes New Infect. 2020; 36:100713. 10.1016/j.nmni.2020.100713 32607246 PMC7293839

[23] Waggoner JJ, Stittleburg V, Pond R, Saklawi Y, Sahoo MK, Babiker A, Hussaini L, Kraft CS, Pinsky BA, Anderson EJ, Rouphael N. Triplex Real-Time RT-PCR for Severe Acute Respiratory Syndrome Coronavirus 2. Emerg Infect Dis. 2020; 26:1633–35. 10.3201/eid2607.201285 32294051 PMC7323516

[24] Jian MJ, Chung HY, Chang CK, Lin JC, Yeh KM, Chiu SK, Wang YH, Liao SJ, Li SY, Hsieh SS, Perng CL, Chang FY, Shang HS. Novel automated sample-to-result SARS-CoV-2 laboratory-developed RT-PCR assay for high-throughput testing using LabTurbo AIO 48 system. Clin Chim Acta. 2021; 514:54–58. 10.1016/j.cca.2020.12.003 33316217 PMC7836538

[25] Lu X, Wang L, Sakthivel SK, Whitaker B, Murray J, Kamili S, Lynch B, Malapati L, Burke SA, Harcourt J, Tamin A, Thornburg NJ, Villanueva JM, Lindstrom S. US CDC Real-Time Reverse Transcription PCR Panel for Detection of Severe Acute Respiratory Syndrome Coronavirus 2. Emerg Infect Dis. 2020; 26:1654–65. 10.3201/eid2608.201246 32396505 PMC7392423

[26] Gong YN, Tsao KC, Hsiao MJ, Huang CG, Huang PN, Huang PW, Lee KM, Liu YC, Yang SL, Kuo RL, Chen KF, Liu YC, Huang SY, et al. SARS-CoV-2 genomic surveillance in Taiwan revealed novel ORF8-deletion mutant and clade possibly associated with infections in Middle East. Emerg Microbes Infect. 2020; 9:1457–66. 10.1080/22221751.2020.1782271 32543353 PMC7473175

[27] Jian MJ, Chung HY, Chang CK, Hsieh SS, Lin JC, Yeh KM, Chen CW, Chang FY, Chiu SK, Hung KS, Liu MT, Yang JR, Perng CL, Shang HS. Investigation of One Familial Cluster of COVID-19 in Taiwan: Differentiation of Genetic Variation Among Isolates and Implications for Epidemiological Investigation and Surveillance by Genomic Assay. Infect Drug Resist. 2021; 14:971–77. 10.2147/IDR.S298451 33737819 PMC7961209

[28] Hadfield J, Megill C, Bell SM, Huddleston J, Potter B, Callender C, Sagulenko P, Bedford T, Neher RA. Nextstrain: real-time tracking of pathogen evolution. Bioinformatics. 2018; 34:4121–23. 10.1093/bioinformatics/bty407 29790939 PMC6247931

[29] Huddleston J, Hadfield J, Sibley TR, Lee J, Fay K, Ilcisin M, Harkins E, Bedford T, Neher RA, Hodcroft EB. Augur: a bioinformatics toolkit for phylogenetic analyses of human pathogens. J Open Source Softw. 2021; 6:2906. 10.21105/joss.02906 34189396 PMC8237802

