# Supplementary material for: Multicenter study evaluating one multiplex RT-PCR assay to detect SARS-CoV-2, influenza A/B, and respiratory syncytia virus using the LabTurbo AIO open platform: epidemiological features, automated sample-to-result, and high-throughput testing
Source: Aging (Albany NY). 2021 Dec 12;13(23):24931–42. doi: 10.18632/aging.203761 (PMC8714143; doi:10.18632/aging.203761)
Supplement: Supplementary Figure 1 [file aging-13-203761-s001.pdf]

SUPPLEMENTARY FIGURE

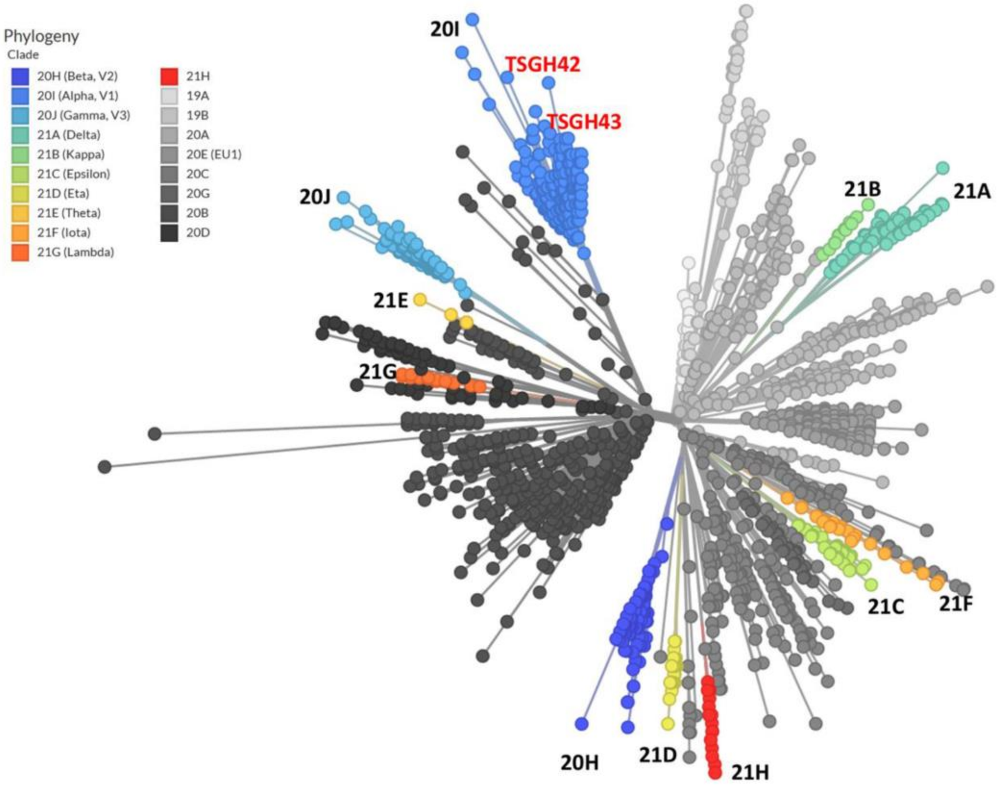

Supplementary Figure 1. The clade of SARS-CoV-2 (TSGH42 and TSGH43) collected form retrospective positive specimens in this study. The clade belongs to SARS-CoV-2 B.1.1.7 variant (20I).
