# Supplementary material for: Multicenter study evaluating one multiplex RT-PCR assay to detect SARS-CoV-2, influenza A/B, and respiratory syncytia virus using the LabTurbo AIO open platform: epidemiological features, automated sample-to-result, and high-throughput testing
Source: Aging (Albany NY). 2021 Dec 12;13(23):24931–42. doi: 10.18632/aging.203761 (PMC8714143; doi:10.18632/aging.203761)
Supplement: Supplementary Tables [file aging-13-203761-s002.pdf]

SUPPLEMENTARY TABLES

Supplementary Table 1. Assessment of Limit of detection for mixed pathogens of respiratory tract virus in multiplex RT-PCR.

Mixed pathogen (RNA copies per PCR)	SARS-CoV-2 RNA copies per PCR (No. of replicates detected at each dilution/total no. of replicates at indicated no. of copies per PCR (percentage))						
	300	75	24	18.8	9.4	3.8	1.9
Influenza A H1 (300)	20/20 (100)	20/20 (100)	20/20 (100)	20/20 (100)	19/20 (95)	11/20 (55)	0/20 (0)
Influenza A H1 (24)	20/20 (100)	20/20 (100)	20/20 (100)	20/20 (100)	19/20 (95)	12/20 (60)	0/20 (0)
Influenza A H3 (300)	20/20 (100)	20/20 (100)	20/20 (100)	20/20 (100)	19/20 (95)	12/20 (60)	0/20 (0)
Influenza A H3 (24)	20/20 (100)	20/20 (100)	20/20 (100)	20/20 (100)	19/20 (95)	10/20 (50)	0/20 (0)
Influenza A H1N1(300)	20/20 (100)	20/20 (100)	20/20 (100)	20/20 (100)	19/20 (95)	12/20 (60)	0/20 (0)
Influenza A H1N1(24)	20/20 (100)	20/20 (100)	20/20 (100)	20/20 (100)	19/20 (95)	11/20 (55)	0/20 (0)
Influenza B (300)	20/20 (100)	20/20 (100)	20/20 (100)	20/20 (100)	19/20 (95)	10/20 (50)	0/20 (0)
Influenza B (24)	20/20 (100)	20/20 (100)	20/20 (100)	20/20 (100)	19/20 (95)	12/20 (60)	0/20 (0)
RSV subtype A (300)	20/20 (100)	20/20 (100)	20/20 (100)	20/20 (100)	19/20 (95)	11/20 (55)	0/20 (0)
RSV subtype A (24)	20/20 (100)	20/20 (100)	20/20 (100)	20/20 (100)	19/20 (95)	12/20 (60)	0/20 (0)
RSV subtype B (300)	20/20 (100)	20/20 (100)	20/20 (100)	20/20 (100)	19/20 (95)	11/20 (55)	0/20 (0)
RSV subtype B (24)	20/20 (100)	20/20 (100)	20/20 (100)	20/20 (100)	19/20 (95)	12/20 (60)	0/20 (0)

Supplementary Table 2. Primers and probe used in this study.

Primer name	Description	Primer sequence (5′→3′)	References
**2019-nCov_N2-F**	2019-nCoV-forward sequence	TTACAAACATTGGCCGCAAA	[25]
**2019-nCov_N2-R**	2019-nCoV reverse sequence	GCGCGACATTCCGAAGAA	[25]
**2019-nCov_N2-P**	2019-nCoV probe	FAM-ACAATTTGCCCCCAGCGCTTCAG-BHQ-1	[25]
**InfA-F**	Influenza A virus forward sequence	CCMAGGTCGAAACGTAYGTTCTCTCTATC	[14]
**InfA-R**	Influenza A virus reverse sequence	TGACAGRATYGGTCTTGTCTTTAGCCAYTCCA	[14]
**InfA-P**	Influenza A virus probe	VIC-ATYTCGGCTTTGAGGGGGCCTG-BBQ	[14]
**InfB-F**	Influenza B virus forward sequence	GAGACACAATTGCCTACTTGCTT	[14]
**InfB-R**	Influenza B virus reverse sequence	TTCTTTCCCACCAAACCAAC	[14]
**InfB-P**	Influenza B virus probe	Cy5-AGAAGATGGAGAAGGCAAAGCAGAACTAGC-BBQ	[14]
**RSV-N-F**	Respiratory syncytial virus forward sequence	CTGTCATCCAGCAAATACAC	[14]
**RSV-N-R**	Respiratory syncytial virus reverse sequence	GCATATAACATACCTATTAAYCC	[14]
**RSV-N-P**	Respiratory syncytial virus probe	Texas red—ACAGGAGATARTATTGAYACTCCYAAT-BBQ	[14]
**RP-F**	RNase P forward sequence	AGA TTT GGA CCT GCG AGC G	[25]
**RP-R**	RNase P reverse sequence	GAG CGG CTG TCT CCA CAA GT	[25]
**RP-P**	RNase P probe	Cy5.5-TTC TGA CCT GAA GGC TCT GCG CG-BBQ	[25]
